# Large-Scale Green Method for Synthesizing Ultralong Uniform Tellurium Nanowires for Semiconductor Devices

**DOI:** 10.3390/nano14201625

**Published:** 2024-10-10

**Authors:** Zhiyi Lyu, Mose Park, Yanjin Tang, Hoon Choi, Seung Hyun Song, Hoo-Jeong Lee

**Affiliations:** 1 Department of Physics, Sungkyunkwan University, 2066, Seobu-ro, Jangan-gu, Suwon-si 16419, Republic of Korea; 2 Department of Smart Fab. Technology, Sungkyunkwan University, Suwon 16419, Republic of Korea; 3 Department of Electrical Engineering, Sookmyung Women’s University, Seoul 04310, Republic of Korea

**Keywords:** tellurium nanowires, green synthesis, semiconductor applications, thin-film transistors, large-scale

## Abstract

This study presents a large-scale green approach for synthesizing ultralong tellurium nanowires with diameters around 13 nm using a solution-based method. By adjusting key synthesis parameters such as the surfactant concentration, temperature, and reaction duration, we achieved high-quality, ultralong Te NWs. These nanowires exhibit properties suitable for use in semiconductor applications, particularly when employed as channel materials in thin-film transistors, displaying a pronounced gate effect with a high switch of up to 10^4^ and a mobility of 0.9 cm^2^ V^−1^s^−1^. This study underscores the potential of solvent-based methods in synthesizing large-scale ultralong Te NWs as a critical resource for future sustainable nanoelectronic devices.

## 1. Introduction

Tellurium (Te) belongs to the VI group of the chalcogen elements and exhibits a tunable bandgap ranging from 0.33 to 1.42 eV based on size and dimension scaling [1,2,3,4,5]. The single element aspect of Te, akin to silicon or carbon-based, suggests that high-quality materials can be grown for semiconductor devices [6,7,8]. While Te can be grown in different dimensions (e.g., nanoparticles, nanowires, nanosheets, etc.), 1-D Te nanowires (Te NWs) hold immense promise for high-performance electronics as they can maintain an accessible bandgap (<2 eV) when scaled down to the nano scale. In addition, ultralong Te NWs can function similarly to carbon nanotubes, thus making Te NWs with controlled dimensions, exceptional mobility, and energy efficiency among the key contenders for extending Moore’s Law [9,10]. However, for industrial applications, it is imperative to devise a technique for the large-scale synthesis and meticulous size regulation of Te NWs for semiconductor devices [11,12]. However, the synthesis of high-quality, size-controlled, uniform, and ultralong Te NWs remains a significant challenge [13]. To overcome this challenge, researchers have proposed various synthesis methods, including chemical vapor deposition (CVD) [14], physical vapor deposition (PVD) [15], microwave-assisted methods [16], the electrochemical deposition method [17], and the hydrothermal method [18,19]. Among these methods, the solution-based approach is widely regarded as the most promising for achieving size-controlled and uniform large-scale production of Te NWs [18]. Nanometers and lengths extending to several micrometers were studied by Furuta [20]. Meanwhile, Li [21] and colleagues successfully synthesized Te NWs with diameters ranging from 72 to 240 nm and lengths, making the bandgap rather small for room-temperature semiconductor applications. Another approach involved a one-pot hydrothermal method to synthesize uniform Te NWs with diameters ranging from 7 to 9 nm; however, the lengths were limited to several micrometers. In addition, this method had drawbacks, such as the unfriendly environment involving hydrazine hydrate and ammonia, along with potential safety concerns associated with high-temperature, high-pressure, large-scale synthesis that required thickened reaction vessels and Teflon linings [19]. Despite the variety of methods proposed for preparing tellurium nanostructures, traditional approaches have often struggled to meet the demand for environmentally friendly, large-scale production of ultralong Te NWs.

In this study, we introduced a synthesis method for ultralong Te NWs that is both environmentally friendly and suitable for large-scale production. We fabricated the proof-of-concept transistor using the synthesized nanowires. Our emphasis was on the solvent-based approach, which is conducted under ambient pressure, making it an eco-friendly method for synthesizing large quantities of ultralong Te NWs. Our research covers a comprehensive investigation into various synthesis parameters, including temperature, reaction time, and the addition of surfactants, in order to gain a deep understanding of their impact on key characteristics of the nanowires, such as crystal structure and diameter, to increase the bandgap significantly. These efforts are geared toward optimizing the synthesis process to obtain scaled dimensions and quantum confinement effects expected to open the bandgap, making them suitable for use as channel materials in thin-film field-effect transistors (TFTs). The outcomes of this research provide a potential key material resource for nanoelectronic devices in the future.

## 2. Materials and Methods

### 2.1. The Chemicals and Materials

All raw materials used in this study were procured from Sigma-Aldrich, Seoul, Republic of Korea and did not require further purification. These materials included TeO_2_ (≥99%) from Sigma-Aldrich, polyvinylpyrrolidone (PVP, average molecular weight 40,000), KOH (≥99%) from Sigma-Aldrich, ethylene glycol from Sigma-Aldrich, and citric acid (≥99%) from Sigma-Aldrich.

### 2.2. Synthesis of Tellurium Nanowires

Preparation of Solution A: In a 10 mL beaker, 3 mL of ethylene glycol was measured. Then, 0.032 mmol of TeO_2_ and polyvinylpyrrolidone (PVP, molecular weight 40,000) were added and mixed. Preparation of Solution B: In a separate 10 mL beaker, 0.3 g of KOH was added to 3 mL of ethylene glycol. The mixture was sealed and stirred under vacuum for 30 min. Preparation of Solution C: In another container, 0.2 g of ascorbic acid was measured and added to 6 mL of deionized (DI) water. The solution was stirred until the citric acid dissolved. Solutions A, B, and C were then degassed under vacuum for 30 min. Solution A was slowly added to solution B under nitrogen protection and heated to 120 °C. Next, 3 mL of the previously heated solution C (at 90 °C) was rapidly added. The reaction was conducted under nitrogen protection at 120 °C for 24 h. Once the synthesis was finalized, the resultant product underwent a meticulous cleansing process involving three rinses with water and three subsequent rinses with alcohol. Following this thorough purification, the material was dried at 60 °C under vacuum conditions for a duration of 6 h. This meticulous procedure ensured the removal of any residual impurities, rendering the nanomaterial ready for subsequent analyses and applications. A 2000 mL sample was prepared with the same proportions as above, with variations in stirring speed (increased from 400 rpm to 1500 rpm) and vacuum evacuation time (2 h of stirring). In this case, solution C was prepared using ethylene glycol instead of DI water and preheated to 120 °C.

### 2.3. Material Characterization

X-ray Diffraction (XRD): XRD patterns were collected using a Bruker-D8 DISCOVER instrument (Bruker, Karlsruhe, Germany). Field Emission Scanning Electron Microscopy (FESEM): FESEM images were obtained using a JSM-7600F microscope (JEOL Ltd., Tokyo, Japan). Raman and Photoluminescence Spectroscopy: Raman spectra were measured using a Witec Alpha 300M+ confocal microscopy and spectroscopy analysis system (Witec, Ulm, Germany). X-ray Photoelectron Spectroscopy (XPS): Elemental information was collected using a Thermo ESCALAB 250 HRXPS instrument (Thermo Fisher Scientific, Waltham, MA, USA). Transmission Electron Microscopy (TEM): TEM and HRTEM images were acquired using a JEOL Ltd. JEM 2100F instrument (JEOL Ltd., Tokyo, Japan). Electrical Measurements: Electrical measurements of the fabricated devices were conducted at room temperature in a dark environment using a Keithley 4200B-SCS Parameter Analyzer semiconductor device analyzer (Keithley Instruments, Solon, OH, USA). Optical Bandgap: This aspect was measured using a UV-VIS-NIR Spectrophotometer, with model from Agilent Technologies (Cary 5000) (Agilent Technologies, Santa Clara, CA, USA).

## 3. Results

**Figure 1:**
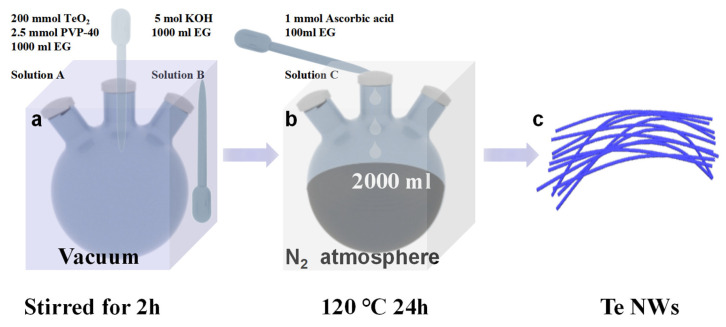
Introduction to the solvent-thermal synthesis method for tellurium nanowires.

The solvent-thermal synthesis method for Te NWs is illustrated in Figure 1. For detailed information, please refer to the Materials and Methods section. In short, solution A and solution B, after vacuum degassing, were reacted at 120 °C under nitrogen protection for 24 h, where the Te NWs are synthesized via reduction of TeO_2_. After the reaction was completed, the resulting product was subjected to three washes with water and three washes with alcohol, followed by vacuum drying for 6 h, and then reserved for further analysis.

**Figure 2:**
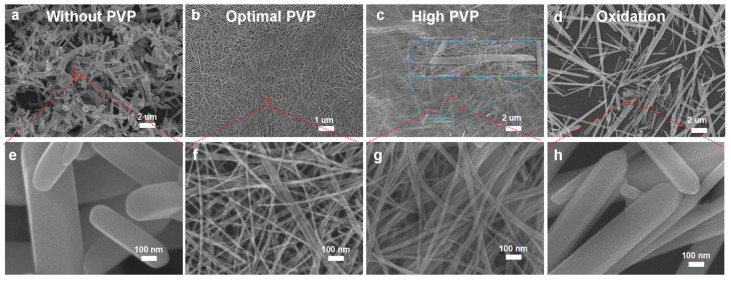
Effect of surface surfactant: (**a**) without surface surfactant, (**b**) with an optimal surface surfactant concentration, (**c**) with double the surface surfactant concentration, (**d**) in an oxygen-rich environment. (**e**), (**f**), (**g**) and (**h**) correspond to high-magnification SEM images of (**a**), (**b**), (**c**), and (**d**), respectively.

The following sections discuss the characterizations and properties of the synthesized nanowires in detail. Firstly, we investigated the effects of the surfactant on the morphology of the grown nanowires. Our results revealed the critical role of the surfactant PVP concentration and the anaerobic environment in achieving uniform Te NWs. To gain a deeper understanding of how PVP affects the dimensionality of Te nanomaterials, we conducted comparative experiments using varying masses of PVP, as shown in Figure 2a–c. In the absence of PVP, both nanowires and nanoparticles were observed. With the addition of surfactant (PVP), however, the formation of the nanoparticles is suppressed, leading to the 1D Te NWs to grow exclusively. We found that, under the optimal conditions of 120 °C for 24 h, Te NWs with relatively uniform diameters of about 13 nm with a length over 100 μm were synthesized (Figure 2b), as highlighted by the red magnification in Figure 2f. When PVP concentration was increased further beyond the optimum, thin nanosheets began to appear, as highlighted by the blue magnification in Figure 2c, and the nanowires shortened to approximately 10 µm (Figure 2c). This was due to the continuous coverage of the surface along the (001) direction by the high concentration of PVP, promoting the growth of 2D tellurium. Additionally, we found that maintaining oxygen-deficient conditions during the synthesis was important in obtaining Te NWs with uniform diameters, potentially due to the oxidation of ascorbic acid by the dissolved oxygen (Figure 2d).

**Figure 3:**
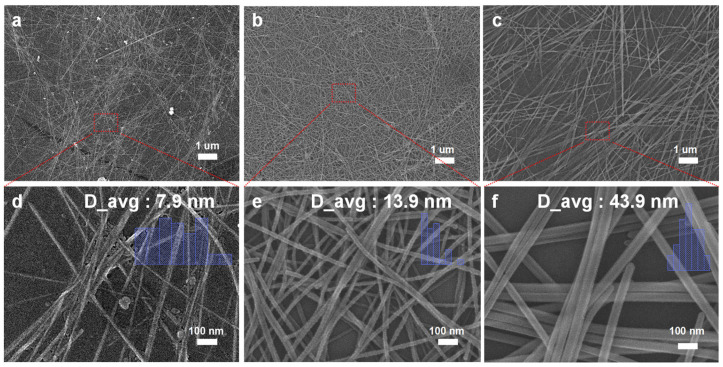
Effect of temperature: (**a**) 90 °C, (**b**) 120 °C, and (**c**) 160 °C. High-magnification SEM images corresponding to (**d**), (**e**), and (**f**), respectively, depict (**a**), (**b**), and (**c**).

Next, we examined the impact of temperature on the growth of nanowires. At 90 °C, as shown in Figure 3a,d, the lower temperature leads to slower nanowire growth with an average diameter of 7.9 nm but the formation of a higher amount of tellurium nanospheres [22]. When the temperature is increased to 120 °C, as seen in Figure 3b,f, better NW growth, with an average diameter of 13.9 nm, and quality are observed, resulting in improved single-crystal growth and longer nanowires. This temperature is more favorable for NW growth compared to 90 °C, leading to an increased longitudinal growth rate and longer nanowires. However, when the temperature is further raised to 160 °C, it can lead to the formation of overgrowth of larger NWs with an average diameter of 43.9 nm. Figure 3 (d), (e), and (f) are magnified images based on Figure 3 (a), (b), and (c), respectively. Each image includes 50 nanowires used to calculate the average diameter. It can be observed that at lower temperatures, tellurium nanowires grow slowly, resulting in the formation of by-products and a sparser distribution. With increasing temperature, the growth rate increases, and a denser network of nanowires is formed. These results emphasize that the synthesis temperature is a critical parameter for controlling the size and morphology of tellurium nanowires. In addition to temperature, the growth time also has a significant impact on the morphology of tellurium nanowires.

**Figure 4:**
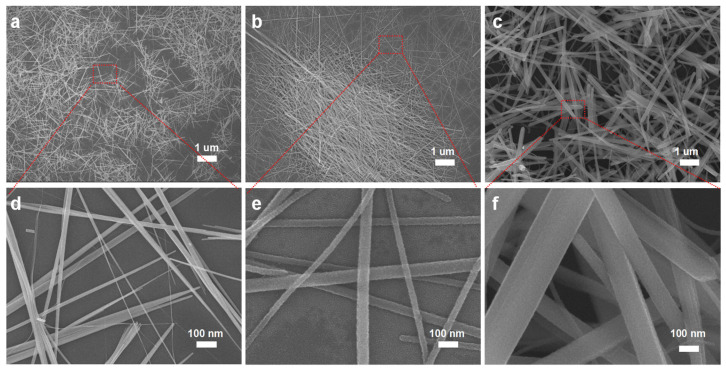
Effect of growth time: (**a**) 2 h, (**b**) 12 h, and (**c**) 48 h. High-magnification SEM images corresponding to (**d**), (**e**), and (**f**), respectively, represent (**a**), (**b**), and (**c**).

Having optimized both the growth environments and temperature, we investigated the Te NW growth as a function of reaction time. In Figure 4, we present SEM images of Te nanostructures synthesized over varying growth durations. Under the 2 h growth condition (Figure 4a), the morphology predominantly exhibits a needle-like shape with a relatively short length. As the growth duration increases, as observed in Figure 4b, the structures evolve into a wire-like form. When the growth duration is further extended to 48 h, as shown in Figure 4c, we observed the development of 2D nanosheets, indicating a transition to growth predominantly along the two-dimensional plane. This indicates that the growth duration is one of the key factors in achieving high-quality Te NWs. Beyond exploring temperature and time, other parameters in amplifying experiments also influence the nanowire morphology. Subsequently, we performed a 1000× magnification on the best-grown samples and conducted in-depth studies on the large-scale synthesized samples.

**Figure 5:**
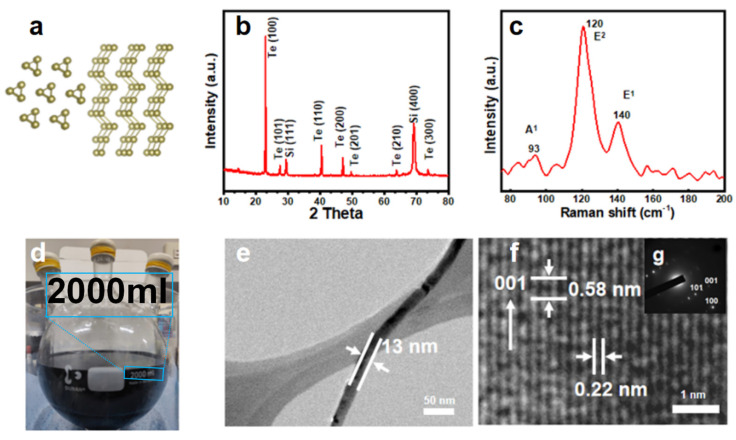
(**a**) Visual lattice diagram of Te nanowires in z and y directions, (**b**) The XRD data, (**c**) Raman spectra, and (**d**) a photograph showing the magnified reaction of a 2000 mL solution of Te NWs. (**e**) displays a TEM image of nanowire. (**f**) is based on the high-resolution TEM image obtained from a single nanowire, (**g**) shows the Selected Area Electron Diffraction (SAED) pattern of the prepared Te nanowires.

Figure 5a depicts a visual lattice diagram of Te nanowires in z and y directions. In Figure 5b, the XRD spectrum of Te NWs synthesized on a large scale is displayed. The distinct diffraction peaks observed at 23.04°, 27.56°, 40.45°, and 47.05° are identified as corresponding to the (100), (101), (102), and (200) crystal planes of Te NWs, respectively. These observations are in accordance with the JCPDS file No. 36-1452. This confirms the successful synthesis of pure Te NWs with (001) preferred growth direction high crystalline. The Raman spectrum of Te NWs is shown in Figure 5b. The peaks at 93 cm^−1^, 120 cm^−1^, and 140 cm^−1^ correspond to the chain extension mode, bond bending, and bond stretching modes [1,23], respectively. These peaks are consistent with previous reports [24]. Figure 5d exhibits a magnified photograph of the reaction involving a 2000 mL solution of Te NWs. Figure 5e displays TEM images of the Te NW a diameter of approximately 13 nm. Additional analysis revealed that the synthesized nanowires had an average diameter of 13.9 nm with a standard deviation of 1.1 nm (n = 50), as shown in Figure 3e. The synthesized product is linear and well-formed. Figure 5f shows a TEM image of the Te NWs, displaying uniformly spaced lattice fringes, indicating excellent crystallinity. The distance between adjacent lattice fringes is approximately 0.58 and 0.22 nm, consistent with the (001) and (110) crystal planes of Te (JCPDS No. 36-1452). This, combined with the electron diffraction pattern of a single Te NW in Figure 5f, demonstrates that the Te NWs grow along the (001) direction, further confirming the preparation of perfect single-crystalline NWs that grow along the (001) direction. PVP plays a crucial role in promoting the non-helical chain-like structure of Te NWs [25], resulting in highly crystalline growth patterns, which are key factors in improving electron transport performance.

**Figure 6:**
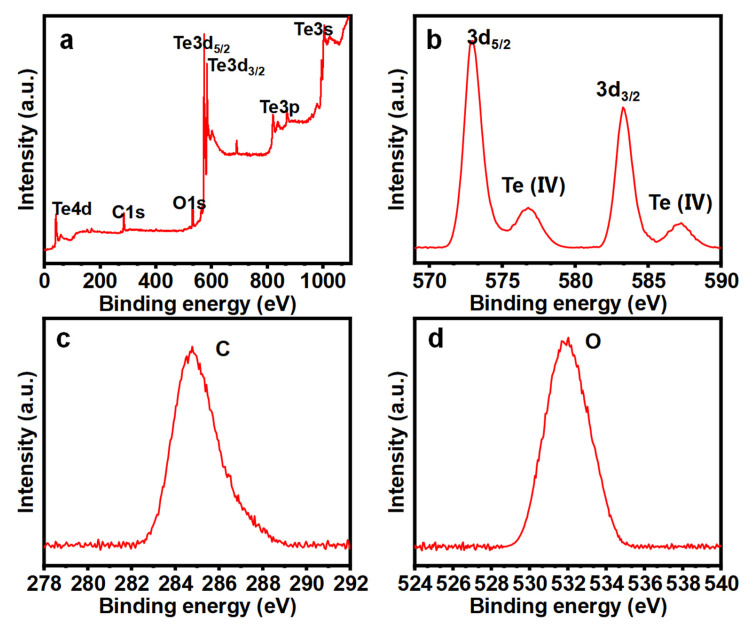
The high-resolution X-ray Photoelectron Spectroscopy (XPS) core-level spectra, (**a**) full spectrum, (**b**) Te-spectrum, (**c**) C-spectrum, and (**d**) O-spectrum.

XPS analysis was conducted to assess the chemical state and composition of the Te NWs. In Figure 6a–b, the XPS spectra of Te NWs are presented. The complete XPS spectrum (Figure 6a) confirms the presence of Te along with small amounts of carbon (Figure 6c) and oxygen (Figure 6d) elements. Figure 6b displays the HR-XPS spectrum of Te 3d, displaying two prominent peaks at 572.9 and 583.4 eV, attributed to Te 3d_5/2_ and Te 3d_3/2_ in Te, respectively [26]. Two additional features observed at higher binding energies (576.1 and 586.4 eV) display Te oxide phases, which are due to the rapid oxidation of Te upon exposure to air, forming a spontaneous oxide layer [27].

**Figure 7:**
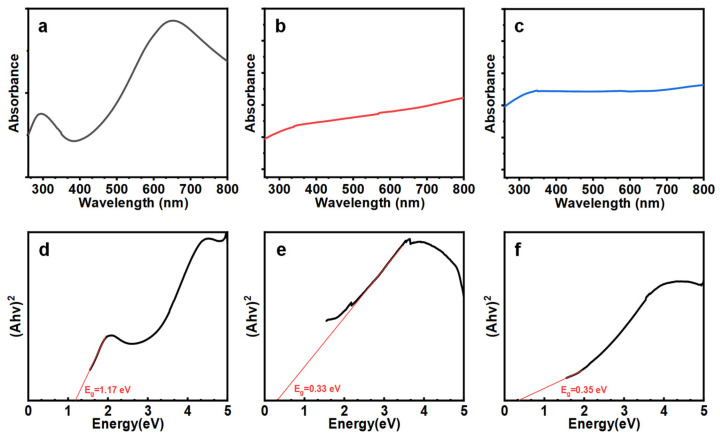
UV-vis spectrum of Te nanowires (**a**) Te NWs grown at 120 °C for 24 h under nitrogen protection, (**b**) Te nanostructure grown without PVP, and (**c**) Te nanostructure grown in an oxygen-containing environment. (**d**), **(e**), and (**f**) are the Tauc plots used to estimate the optical bandgap for (**a**), (**b**), and (**c**), respectively.

Next, we investigated the bandgap of the Te NWs with different diameters to confirm that the dimension control is critically related to the diameter of the wires. We adopted the UV-Vis absorption spectrum to estimate the optical bandgap of the nanowires. As shown in Figure 7a, there is a strong absorption peak at 680 nm, which is not found in Figure 7b,c. Figure 7 (d), (e), and (f) are Tauc plots used to estimate the optical bandgap for (a), (b), and (c), respectively. The results in Figure 7d indicate that the bandgap for nanowires with a diameter of 13 nm is 1.17 eV, wider than the optical bandgap of bulk materials shown in Figure 7e at 0.33 eV and Figure 7f at 0.35 eV. This widening is due to quantum confinement effects and is consistent with previous theoretical calculations [1]. The significant increase in the bandgap addresses the main drawback of Te as a material for future ultra-large-scale integration transistor channels. These findings emphasize the potential of thin and long Te NWs for high-performance semiconductor applications.

**Figure 8:**
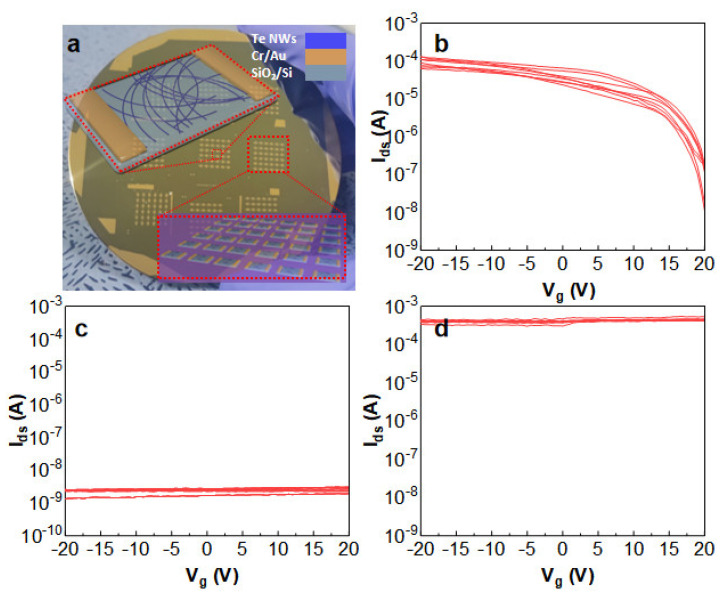
(**a**) The image shows Te NW TFT devices. (**b**) I/V Logarithmic plot of the transfer characteristics at Vds = −20 V for Te NWs with a diameter of 13 nm. (**c**) I/V Logarithmic plot of the transfer characteristics at Vds = −20 V for short and thick Te NWs samples. (**d**) I/V Logarithmic plot of the transfer characteristics at Vds = −20 V for thick and long Te NWs samples.

Furthermore, nanowire thin-film transistors (TFTs) leverage these nanowires as channel materials and operate based on the field-effect principle. As shown in Figure 8a, the typical Te NWs thin-film transistor, aluminum metal electrodes, are patterned on a 2000 Å SiO_2_/Si substrate using thermal evaporation through a stainless steel mask. A 5 mg/mL different morphology (Figure 8b–d) Te NW aqueous solution (1 µL) is drop-cast between the electrodes. The device performance is tested using a semiconductor parameter analyzer under ambient temperature and dark conditions. Critical parameters, such as the on/off ratio, current ratio, and mobility, were evaluated [28]. With a width of 50 µm and a gate length of 1000 µm, the calculated mobility of Figure 8b (denoted by μ) [29] is approximately 0.9 cm^2^/V·s at Vds = 10 V. While the long and narrow wires showed a clear transistor behavior, the same fabricated with short and thick Te NWs (corresponding to the sample shown in Figure 2a) were mostly insulating, likely due to the poor percolation due to the short wire length. On the other hand, the device fabricated with the overgrown samples (corresponding to Figure 2d) showed high conductivity with minimal gate voltage controllability due to the good percolation, which was due to the long wire lengths and small bandgap due to the large wire diameter. This observation underscores the correlation between semiconductor performance, diameter (related to the bandgap), and length (related to the percolation) when fabricating Te NW-based TFT devices.

## 4. Conclusions

The synthesis of ultralong Te NWs using a green hydrothermal method holds great promise for the development of future nanoscale electronic devices. The use of environmentally friendly solvents and low-temperature synthesis conditions makes this method environmentally friendly and suitable for producing high-quality Te NWs. The properties of Te NWs, such as crystal structure, diameter, and surface area, can be controlled and optimized by carefully adjusting synthesis parameters like temperature, time, and the addition of surfactants. These optimized wafers can be utilized as channel materials in TFTs, where they exhibit promising semiconductor characteristics like high carrier mobility and a high on/off ratio. In summary, the green solution-based synthesis of ultralong Te NWs and their semiconductor properties present new opportunities for the development of high-performance nanoelectronic devices. Further research is necessary to comprehensively understand and optimize the characteristics of Te NWs and their applications in thin-film transistors.

## Data Availability

Data is contained within the article.
